# Rapid turnover of sex chromosomes is associated with speciation in Neotropical armored catfish *Harttia* (Siluriformes, Loricariidae)

**DOI:** 10.1186/s13062-026-00994-y

**Published:** 2026-10-21

**Authors:** Francisco de Menezes Cavalcante Sassi, Fernando Henrique Santos de Souza, Manolo Fernandez Perez, Foyez Shams, Tariq Ezaz, Geize Aparecida Deon, Thomas Liehr, Luohao Xu, Marcelo de Bello Cioffi

**Affiliations:** 1 Aquaculture Center of São Paulo State University (CAUNESP), Jaboticabal, 14884-900 SP Brazil; 2 Laboratory of Evolutionary Cytogenetics, Universidade Federal de São Carlos, São Carlos, 13565-905 SP Brazil; 3 Real Jardín Botánico – RJB-CSIC, Madrid, 28014 Spain; 4 Institute for Applied Ecology, University of Canberra, Canberra, ACT 2617 Australia; 5 Jena University Hospital, 07747 Jena, Jena, Germany; 6 MOE Key Laboratory of Freshwater Fish Reproduction and Development, College of Life Sciences, Southwest University, Chongqing, 400715 China

**Keywords:** SNP, Phylogenetics, DArT-seq, Sex-linked markers, Probabilistic modeling

## Abstract

**Background:**

The Neotropical armored catfish genus *Harttia* is an exceptional model for studying sex chromosome evolution, as it harbors the highest diversity of multiple sex chromosome systems known among fishes. However, the evolutionary history of these systems and their contribution to lineage diversification remain unresolved. We generated genome-wide DArTseq data for 17 *Harttia* species to investigate the origins of sex chromosome turnover, reconstruct phylogenetic and biogeographic history, identify sex-linked markers, and test whether multiple sex chromosomes are associated with increased speciation.

**Results:**

Genome-wide SNP analyses resolved three major *Harttia* clades and dated their diversification to the last 10 million years, coinciding with major Neotropical drainage rearrangements. We identified 443 candidate sex-linked markers that suggest independent origins for the X₁X₂Y and XY₁Y₂ multiple sex chromosome systems. Ancestral chromosome-number reconstruction supported an ancestral karyotype of 2n = 58, while integration of candidate sex-linked markers with previous cytogenetic evidence is consistent with an ancestral homomorphic XY system followed by independent chromosome rearrangements generating multiple sex chromosomes in distinct lineages. State-dependent diversification analyses revealed significantly higher speciation rates in clades carrying multiple sex chromosomes than in those with simple systems, suggesting a close association between rapid sex chromosome turnover and lineage diversification.

**Conclusions:**

Our results provide the first genome-wide evolutionary framework for *Harttia*, suggesting that multiple sex chromosome systems may be associated with elevated diversification in some *Harttia* lineages, while highlighting the need for broader genomic and taxonomic sampling to test this association. These findings identify *Harttia* as a powerful model for investigating how sex chromosome turnover, chromosome evolution, and landscape history jointly shape biodiversity.

**Supplementary Information:**

The online version contains supplementary material available at https://doi.org/10.1186/s13062-026-00994-y.

## Background

In the canonical model of sex chromosome evolution, the emergence of a sex-determining gene is followed by the suppression of recombination around this locus, allowing the accumulation of sexually antagonistic alleles and other sex-linked sequences [1, 2]. Structural rearrangements of chromosomes, such as inversions, might further consolidate this recombination suppression, resulting in the gradual differentiation and heteromorphy of the sex chromosomes [3]. Nonetheless, before these chromosome rearrangements in regions where recombination persists, such as the pseudoautosomal region (PAR), sex-linked loci may still recombine between the X and Y or Z and W chromosomes [4]. These dynamics are particularly relevant in species exhibiting homomorphic sex chromosomes, where recombination suppression may be either partial or recent [5]. Comprehending the inheritance patterns, chromosomal locations, and evolutionary trajectories of sex-linked markers is crucial for elucidating the origins and diversity of sex-determination systems [4, 6].

Multiple sex chromosome systems originate from chromosomal rearrangements that occur in simple systems (XX/XY or ZZ/ZW). These rearrangements may involve fissions of ancestral chromosome pairs or fusions with autosomes and are generally considered rare in eukaryotes [7–10], found in only about 100 fish species [11]. However, our understanding of the evolution of sex chromosomes remains largely shaped by studies conducted in a limited number of model organisms, which typically possess heteromorphic sex chromosomes at advanced evolutionary stages, thus providing a restricted view of the diversity and complexity of sex determination systems found in nature [4].

**Fig. 1:**
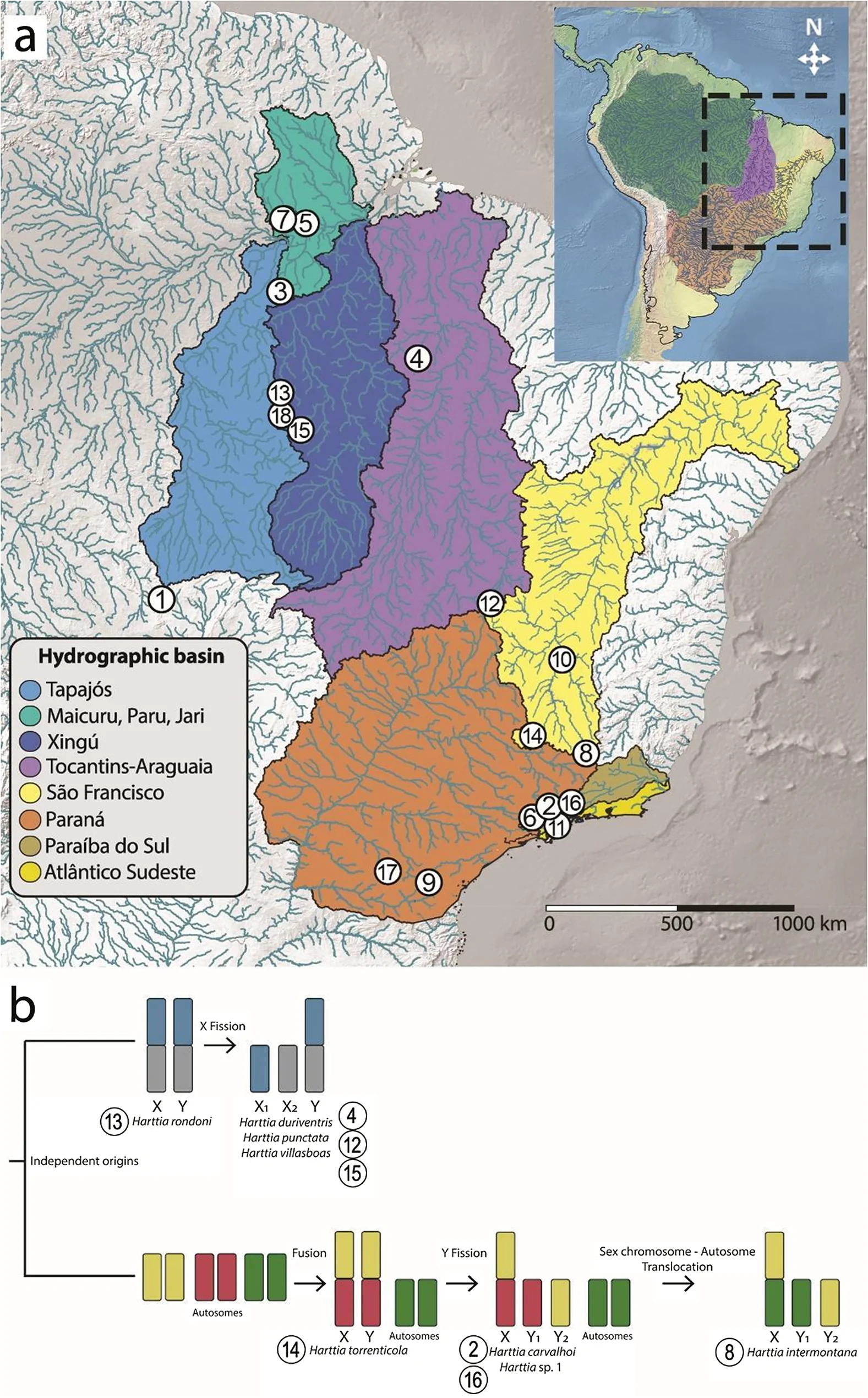
Geographical distribution and sex chromosome evolution pathways of the investigated species**. (a)** Partial Brazilian map indicating sample sites (circles) and river basins (colored areas). Numbering follows Supplementary Table 1. The upper right corner map shows the Neotropical region (black contour line) and the four main basins: Amazon (dark green), Tocantins-Araguaia (purple), La Plata (orange), and São Francisco (yellow). **(b)** Schematic view of the proposed evolutionary pathways of *Harttia* sex chromosomes, based on previous cytogenetic data [18–28]

In this context, the Neotropical armored catfish *Harttia* (Siluriformes: Loricariidae) is emerging as an exceptional model system to study multiple sex chromosomes with distinct degrees of degeneration. Comprising 28 valid species [12], this monophyletic genus [13, 14] is distributed across rheophilic habitats of the Brazilian and Guiana Shields. *Harttia* exhibits the highest number of multiple sex chromosome systems among fish, with about 28% of species displaying heteromorphic sex chromosomes [15], a proportion matched only by *Notobranchius* killifishes [15–17]. These systems vary in complexity and differentiation, making *Harttia* a unique model for studying repeated sex chromosome turnover and the independent origins of multiple sex chromosome systems. Conventional and molecular cytogenetic data revealed an exceptional diversity of 2n (from 52 to 62 chromosomes) and two independent male-heterogametic systems (Fig. 1): the XX/XY_1_Y_2_ and its homologous XX/XY in species from South and Southeast Brazil, and the X_1_X_1_X_2_X_2_/X_1_X_2_Y system with a corresponding XX/XY in Amazonian species [18–21]. In the South and Southeast Brazilian lineage, the ancestral XX/XY configuration represented by *H. torrenticola* is proposed to have undergone a sex chromosome–autosome translocation, generating an intermediate XY configuration. A subsequent rearrangement involving the Y chromosome, interpreted as a Y-chromosome fission associated with an additional autosomal pair, then gave rise to the XY_1_Y_2_ system observed in *H. intermontana* [18, 21–23]. The rare XX/XY_1_Y_2_ system, described for only nine fish species to date [21], is found in three *Harttia* species: *H. carvalhoi* and *Harttia* sp. 1. Conversely, in the Amazonian lineage, a fission of the ancestral XY system (likely the same as seen in *H. rondoni*) is proposed to have led to the emergence of the X_1_X_2_Y system found in *H. duriventris*, *H. villasboas*, and *H. punctata* [24]. Comparative analyses of satellite DNA content reveal distinct degrees of lineage-specific differentiation among these sex chromosome systems, consistent with divergent evolutionary trajectories following their respective chromosomal rearrangements [25, 26].

Without the availability of a reference genome, complexity reduction techniques have proven to be powerful tools for genomic research in non-model organisms [29–31]. These approaches offer a cost-effective means of generating thousands of molecular markers, suitable for investigating genetic polymorphisms, assessing population structure, and exploring patterns of genome evolution [32]. Analyses using large nuclear marker datasets provide higher resolution and assist in overcoming issues such as incomplete lineage sorting and restricted taxon sampling [33, 34]. DArTseq (Diversity Arrays Technology sequencing) [35] has emerged as a widely used method due to its ability to target low-copy and predominantly active genomic regions, which may include sex-specific loci, before high-throughput sequencing [36]. These features have enabled DArTseq applications in a broad range of taxa and research areas, including genetic diversity, hybridization, admixture, phylogeography, phylogenetics, and the identification of sex-linked markers [37–42]. Sex-linked markers provide key insights into the localization and structure of sex-determining regions, offering a molecular entry point into the evolutionary processes that shape sex chromosome systems [37].

In this current study, we performed DArTseq in 17 *Harttia* species and one outgroup species (*Farlowella* sp.) to examine the genomic architecture of sex chromosome evolution in this dynamic genus (Supplementary Table 1). Our primary objective was to identify the likely mechanisms underlying the diversity of multiple sex chromosomes in the genus. To this end, we focused on identifying candidate sex-linked markers and analyzing their distribution across species with different sex chromosome systems. A comprehensive phylogenetic tree based on SNP data was constructed, integrating spatial and cytogenetic information by reconstructing ancestral distribution areas and modeling chromosome number evolution using probabilistic approaches. Our findings indicate that the rapid turnover of multiple sex chromosome systems, originating from an ancestral XY configuration, may be linked to an accelerated tempo of diversification in *Harttia.*

## Results

### Evolutionary relationships in *Harttia*

**Fig. 2:**
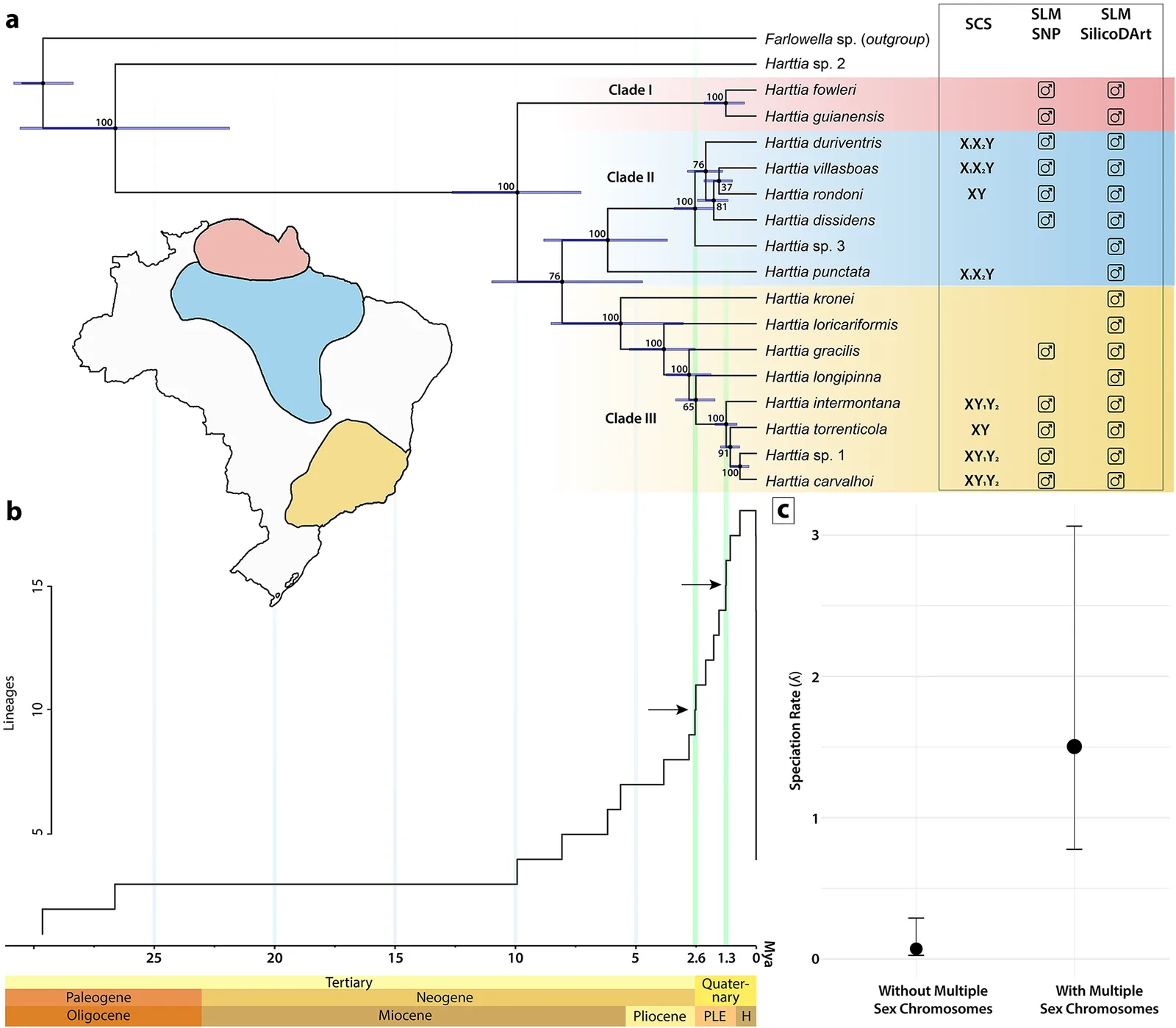
Evolutionary relationships in *Harttia* and the potential role of multiple sex chromosomes in accelerating speciation rates. (**a**) Time-calibrated maximum likelihood phylogenetic relationships of *Harttia* species, using *Farlowella* sp. as an outgroup, recovered from 1,381 autosomal SNPs per species (24,858 SNP markers in total) generated by DArTSeq from 108 individuals. Clade colors correspond to their distribution indicated in the map. Cytogenetically described sex chromosome systems (SCS) are indicated. Sex-linked markers (SLMs), either SNPs or SilicoDArT, are presented using the male symbol to indicate male heterogamety (XY). Node bars correspond to the 95% HPD interval of divergence times, and values at nodes indicate bootstrap support. (**b**) Lineage through time plot, with arrows indicating major shifts in the number of lineages at 2.6 and 1.3 Mya, accompanied by green lines indicating their coincidence with the rapid diversification of clades with multiple sex chromosomes in the phylogeny. The time scale is the same for both the phylogeny and the lineage through time plot. PLE stands for Pleistocene and H for Holocene. (**c**) Speciation rates in clades without and with multiple sex chromosomes inferred by BiSSE. The dots represent the main estimation, and the lines are the 95% confidence intervals

A total of 95,305 SNPs and 282,275 SilicoDArT (presence and absence) loci were generated for 107 individuals from 17 *Harttia* and one *Farlowella* species. The resulting phylogeny, constructed using 24,858 SNP markers after stringent filtering, grouped all *Harttia* species into three primary clades, each exhibiting strong geographic coherence (Fig. 2a). Exceptionally, *Harttia* sp. 2 was placed as a single ancestral branch to all remaining *Harttia*, not included in any of the three clades. Clade I comprises species from the Guiana Shield drainages (*H. fowleri* and *H. guianensis*). At the same time, Clade II included species from Amazon Brazilian Shield drainages, specifically the Xingu, Tapajós, and Tocantins-Araguaia River Basins (*H. duriventris*, *H. dissidens*, *H. punctata*, *H. rondoni*, *H. villasboas*, and *Harttia* sp. 3); and Clade III contained species distributed across Southeastern and South Atlantic drainages and from the La Plata basin (*H. carvalhoi*, *H. torrenticola*, *H. intermontana*, *H. longipinna*, *H. gracilis*, *H. loricariformis*, *H. kronei*, and *Harttia* sp. 1) (Fig. 2a). The divergence between *Harttia* and *Farlowella* was estimated at ~ 29.5 million years ago (Mya), with Clade I split about 10 Mya and the divergence between Clades II and III taking place at ~ 7.9 Mya. The origin of multiple sex chromosome systems was inferred at 5.8 Mya for Clade II (X_1_X_2_Y system, based on the splitting of *H. punctata*) and at 1.3 Mya for Clade III (XY_1_Y_2_ system, based on the splitting of *H. intermontana*). The Principal Component Analysis (PCA) of autosomal SNPs (PC1 = 28.5%, PC2 = 18.9%) identified five distinct clusters aligned with phylogenetic clades and geographic distributions (Supplementary Fig. 2), categorizing *Harttia* species into three primary clusters, while *Harttia* sp. 2 and the outgroup *Farlowella* sp. constituted separate groups. The complete phylogeny with all individuals is presented in Supplementary Fig. 1, and complete mean time-calibration estimates are provided in Supplementary Table 2.

Examining the number of lineages over time reveals two significant shifts (Fig. 2b, arrows). These shifts overlap with splitting times that are mostly related to clades with the presence of multiple sex chromosomes, at 2.5 Mya and 1.3 Mya (i.e., the first for the clade composed of *Harttia* sp. 3, *H. duriventris*, *H. rondoni* and *H. villasboas*, and the second for the clade *H. intermontana*, *H. torrenticola*,* H. carvalhoi*, and *Harttia* sp. 1 (Fig. 2a, b). Indeed, state-dependent diversification analyses with the Binary State Speciation and Extinction model (BiSSE) revealed substantial differences in speciation rates associated with the presence or absence of multiple sex chromosomes. Lineages with simple sex chromosomes (state 0) exhibited a markedly lower speciation rate (λ_0_ = 0.032, CI95% = 0.026–0.290) than those with multiple sex chromosomes (state 1; λ_1_ = 0.941, CI95% = 0.485–3.064). The turnover rate for state 0 (ε = 1.040, CI95% = 0.776–1.230), calculated as the ratio between extinction and speciation, suggests that extinction is approximately equal to speciation in this group. Additionally, the estimated transition rate (q₀↔₁) from the absence to the presence of multiple sex chromosomes indicates a moderate probability of trait evolution over time (0.375, CI95% = 0.202–1.530). These modeling results suggest an association between the presence of multiple sex chromosomes and increased diversification rates in *Harttia* (p-value = 0.005) (Fig. 2c; Table 1).

**Table 1:** Parameter estimates from the BiSSE model evaluating the association between the presence of multiple sex chromosomes and diversification rates. Speciation rates (λ) are reported separately for lineages without (state 0) and with (state 1) multiple sex chromosomes. Turnover rate (ε) corresponds to the extinction/speciation ratio for state 0. The transition rate (q₀→₁) reflects the rate of evolution from the absence to the presence of multiple sex chromosomes. Estimates are presented alongside their 95% confidence intervals

Parameter	Estimate	95% CI
Speciation in clades without multiple sex chromosomes (λ_0_)	0.032	0.016–0.141
Speciation in clades with multiple sex chromosomes (λ_0_)	0.941	0.485–1.947
Turnover (ε₀)	1.040	0.858–1.243
Transition rate (q_0→1_)	0.248	0.135–0.811

**Fig. 3:**
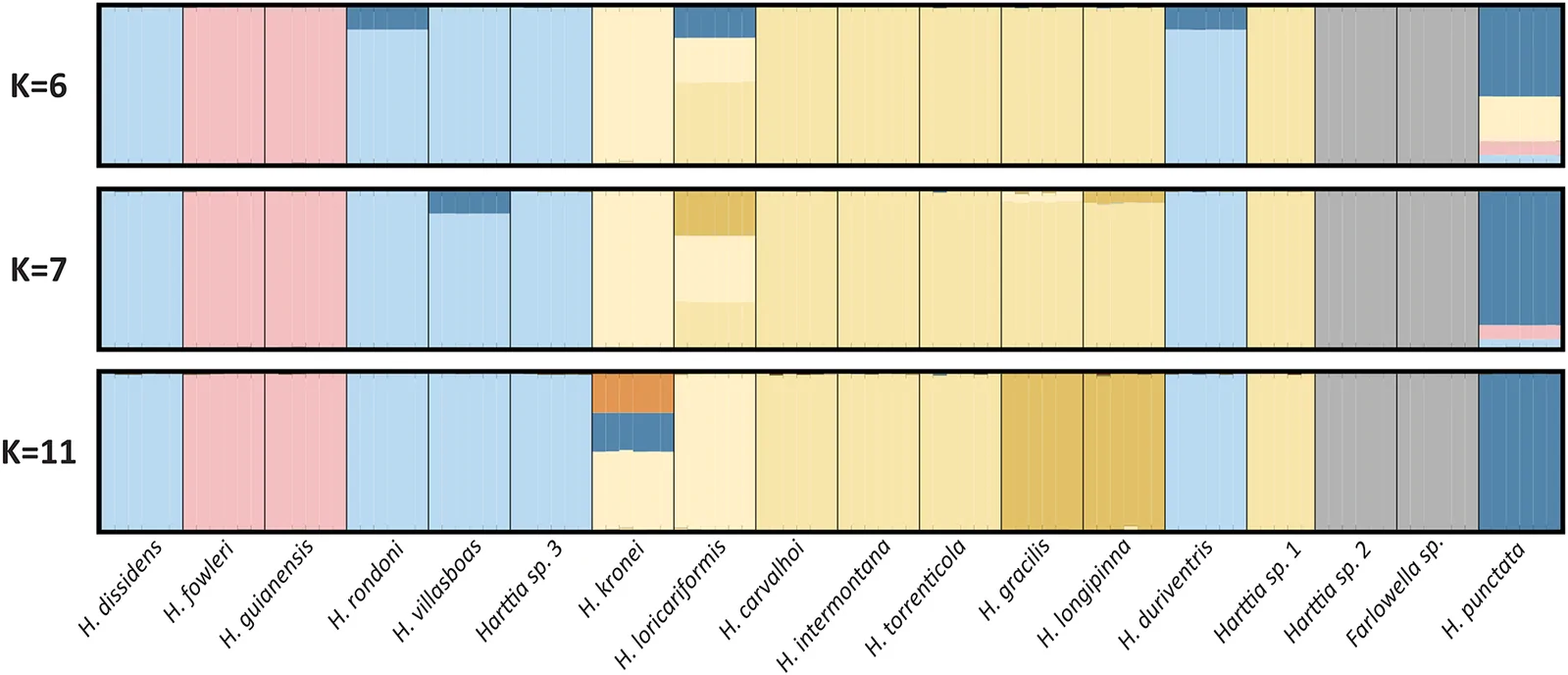
Genetic structure of *Harttia*. Each vertical bar represents an individual, with colors representing the proportion of shared ancestry

The structure plots for *Harttia* are highly congruent with the phylogenetic inference and overall clade organization (Fig. 3). According to the Evanno method, K = 6 best describes the structure, while the ChooseK method is pointed to K = 7 as the best, and the Puechmaille estimator MaxMedK indicated K = 11. Clade 1 consistently appears preserved, as well as the group formed by *Farlowella* and *Harttia *sp. 2. Clade 2 presents a subdivision, separating *H. punctata* from the other species. Clade 3 is the most complex one, with *H. kronei* separating from the other species at K = 6 and three subgroups forming at K = 11 (all according to phylogenetic proximity). There are subtle signs of shared ancestry across clusters; however, these do not seem consistent across different values of K and should not be interpreted as evidence of extensive admixture among the sampled species. Instead, it likely reflects shared ancestry or the effects of incomplete lineage sorting (Fig. 3). It is also relevant to point out that the STRUCTURE standard admixture model assumes Hardy–Weinberg equilibrium within inferred clusters, which is not completely valid for a species dataset.

### Sex-linked markers

The application of a Bonferroni correction for multiple testing across the 95,305 SNP markers generated from the sequencing plate resulted in a stringent per-test significance threshold (P_corrected ≈ 2.62 × 10⁻⁶). This low threshold indicates that the identified sex-linked SNPs are unlikely to be false positives arising from random assortment. We focused the false discovery rate calculation on the SNP data due to its co-dominant nature, which provides higher confidence in genotyping calls for assessing sex-linked patterns rather than dominant SilicoDArT markers. Nevertheless, we acknowledge the intrinsic limitations of our sample size (*n* = 3 per sex) and recognize that a greater sampling is required to provide better statistical confidence for the SLM.

**Fig. 4:**
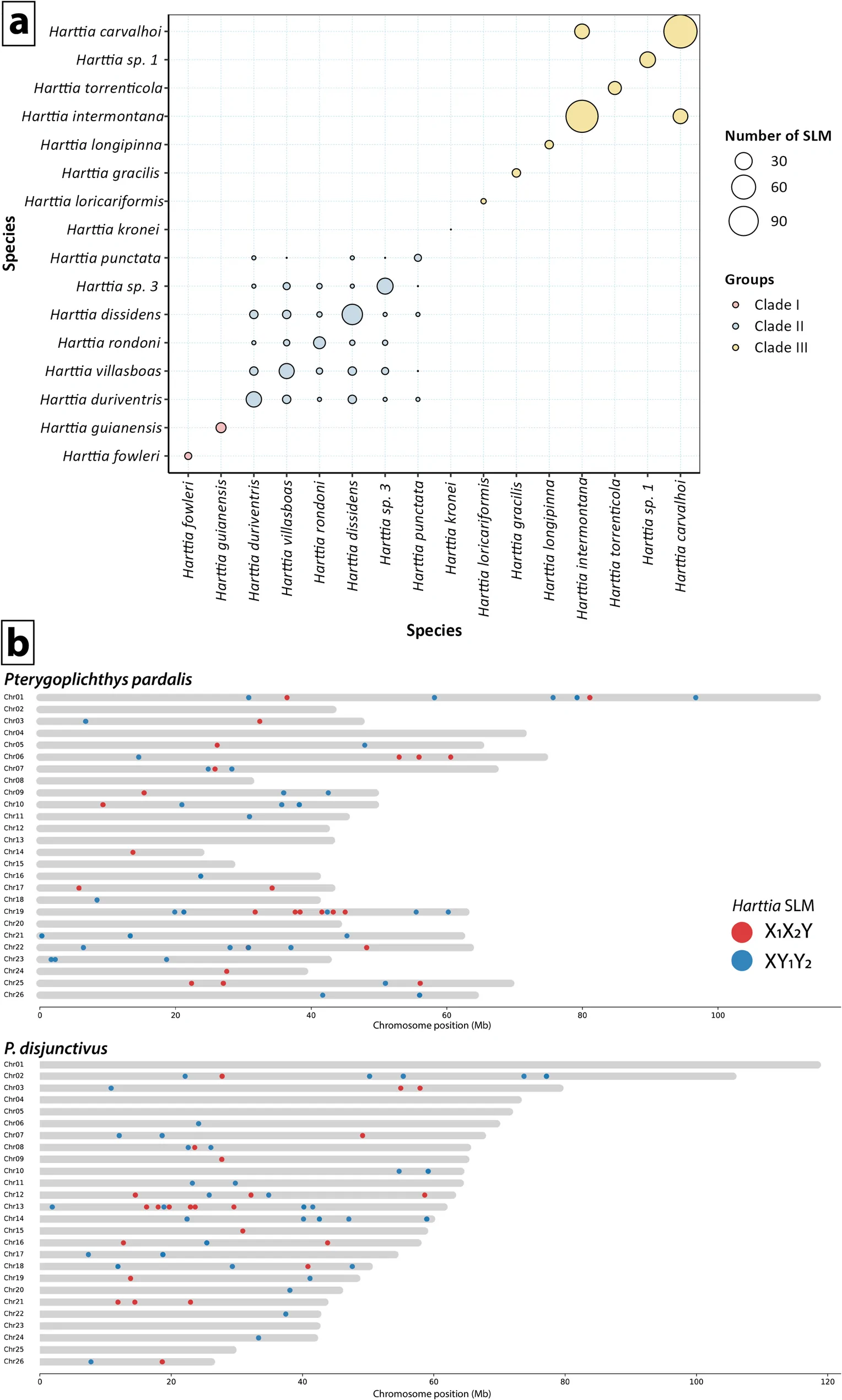
Independent evolution of sex chromosomes in *Harttia* underscored from sex-linked markers. **(a)** Bubble plot showing the number of sex-linked markers per species, colored according to the clade they belong to, as shown in Fig. 2. The off-diagonal bubbles represent shared SLMs. **(b)** Cross-mapping of *Harttia* sex-linked markers in the chromosome-level genome of *Pterygoplichthys pardalis* and *P. disjunctivus* (Loricariidae, Hypostominae)

Across all samples, we identified 443 candidates of sex-linked markers: 95 XY and 16 ZW markers from the SNPs dataset and 316 XY and 16 ZW markers from the SilicoDArT dataset (Fig. 4a; Supplementary Data 1 and 2 available at https://github.com/sassichico/harttia). PCA of the sex-linked SNPs (Supplementary Fig. 2) revealed three main clusters: one composed solely of *H. intermontana*, a second grouping *H. carvalhoi*, *Harttia* sp. 1, and *H. torrenticola*, and a third containing the remaining species, with a minor subcluster of *H. gracilis*. Using a 100% similarity threshold, we identified three shared sex-linked SNPs and 33 shared SilicoDArT markers (Supplementary Table 3). Shared sex-linked markers were detected in two groups: (i) *H. carvalhoi* and *H. intermontana* (XY_1_Y_2_ system) and (ii) a subset of species within Clade II, all in both groups corresponding to XY SLM. Those shared SLMs were only identified inside the groups and not between them, suggesting independent origins of those systems. To further check the reliability of the sex-linked markers, we cross-mapped the SLM we obtained in the two available chromosome-level genomes for Loricariidae at NCBI (*Pterygoplichthys pardalis*, and *P. disjunctivus*), since *Harttia* species do not have assembled genomes. Although these genomes lack an identification of sex chromosomes [43], sex-associated candidate markers were successfully mapped to chromosomes in both *Pterygoplichthys* species, although their distribution differed markedly between assemblies. In *P. pardalis*, markers were concentrated on a subset of chromosomes, with particularly conspicuous clustering on Chr01, whereas in *P. disjunctivus* they were more broadly distributed across the chromosome complement. Markers associated with the XY_1_Y_2_ system were detected on multiple chromosomes in both species rather than being restricted to a single chromosome. Overall, the distribution shows that sex-associated sequences found in *Harttia* are conserved across multiple chromosomal regions in *Pterygoplichthys*, with no clear evidence of a single conserved sex-linked region between the species (Fig. 4b).

### Biogeographical modeling and ancestral karyotype

A key objective of this study was to infer the historical processes underlying the current distribution and diversity of *Harttia*. We therefore employed biogeographical modeling to estimate ancestral ranges and identify potential dispersal routes, coupled with ancestral chromosome number reconstruction to trace the role of chromosome number evolution in speciation. Resolving these questions is fundamental to testing hypotheses about the drivers of diversity in this widely distributed genus. The DIVALIKE model was selected as the best-fitting model based on AICc scores (Table 2; Fig. 4a). Although likelihood scores between the DEC and DIVALIKE models showed minimal variation, the estimated ancestral ranges diverged notably after the 23 Mya time slice (see Supplementary Fig. 4 for DEC estimates). ΔAIC values indicated only a marginal improvement for DIVALIKE over DEC, with the same occurring for Akaike weights (Wi), indicating some uncertainty between them. Both models converged on similar ancestral area reconstructions after the 10 Mya threshold. The most inclusive *Harttia* clade was inferred to have occupied either the Southern or Southeastern Atlantic drainages (A). The most recent common ancestor of Clade III was likely restricted to the “A”, whereas Clade II appears to have been confined to the Amazon Brazilian Shield (B). DIVALike suggested an ancestral distribution along the “A” for the whole *Harttia* clade, with the ancestor of *Harttia* and *Farlowella* likely ranging either on the “A” or “B”.

**Table 2:** Models implemented in BioGeoBEARS with their respective Log-likelihood (lnL), dispersal (d), and extinction (e) parameters, corrected Akaike information criterion (AICc), difference in AICc values compared to the best-fit model (ΔAIC), and Akaike weights (W_i_)

Model	lnL	d	e	AICc	ΔAIC	Weight (W_i_)
DEC	-39.28528	0.0268696	0.0304716	82.59056	0.97624	0.38
DIVALike	-38.80716	0.02924266	0.02305116	81.61432	0	0.62
BayAreaLike	-45.32449	0.03725671	0.08521665	94.64898	13.03466	0.0009

**Fig. 5:**
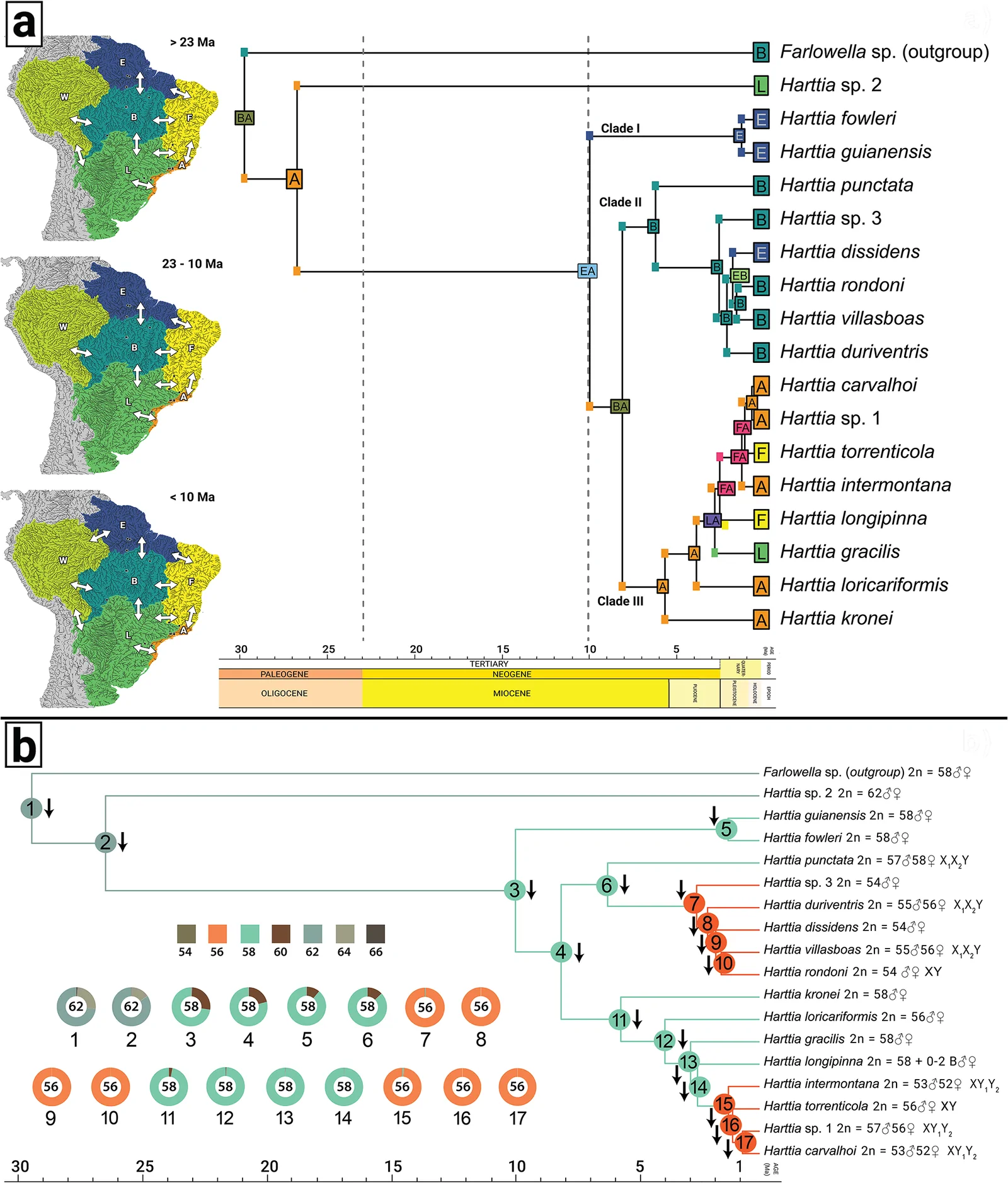
Biogeographic and karyotype modeling for the ancestral characters of *Harttia*. **(a)** Ancestral range of *Harttia* estimated from the best DIVALike model in BioGeoBEARS. Dashed lines correspond to the three time slices defined for modeling: > 23 Mya, 23 − 10 Mya, and < 10 Mya, which are also represented in the maps with the connections allowed at each (white arrows). The color and letters of tips in phylogeny correspond to the same color in the map on the left, representing the biogeographical areas. At the nodes, the letters represent the sum of the areas of the tips, colored by their mean color. **(b)** The evolution of diploid numbers in *Harttia* was inferred in ChromEvol v2.0 with the Dys model. Each branch is colored the same as indicated in the legend for its respective ancestral 2n. Pie charts indicate the probability of the ancestral 2n recovered for each node, as indicated by the numbers (1–17) in the nodes of the phylogenetic tree. The diploid number and sex chromosome system of the species are shown after their names. Chromosome loss events (down arrows) predominated (Supplementary Data 3)

The model Dys proposes that the ancestral 2n at the root of the phylogeny was 2n = 62, indicating that events of descending dysploidy (chromosome fusions) predominated throughout the chromosomal evolution of *Harttia.* For the *Harttia* clade (excluding *Farlowella*), the same ancestral 2n was also found. In clade III, the ancestral diploid number for species exhibiting cytogenetically identifiable sex chromosomes (*H*. *intermontana*, *H*. *torrenticola*, *H*. *carvalhoi*, and *Harttia* sp. 1) is 2n = 56, while in clade II (the most comprehensive clade, including *H. punctata*, *H. duriventris*, and *H. villasboas*), it is 2n = 58 (Fig. 5b, Supplementary Data 3 available at https://github.com/sassichico/harttia).

## Discussion

Our study suggests that the diversification of *Harttia* is likely driven by a combination of biogeographical events, karyotype restructuring, and multiple independent origins of sex chromosome systems. Our phylogenetic reconstruction suggests that *Harttia* diversification occurred primarily over the past 10 million years, coinciding with major geological and hydrological changes in South America. Notably, the emergence of multiple sex chromosome systems that occurred during ~ 5.6 and ~ 1.2 Mya is associated with accelerated speciation rates, as indicated by BiSSE modeling, suggesting an important role of these systems in the evolution and speciation of the genus. These findings reshape our understanding of the genus and present *Harttia* as a model for studying rapid and recurrent sex chromosome turnover and its putative role in speciation.

The nuclear-based phylogeny, based on 1,381 SNPs per species (24,858 SNP markers in total), resolves three major *Harttia* clades but differs from previous mitochondrial and morphological studies. While broadly consistent with earlier studies [13, 14], our topology reveals key discrepancies, such as the placement of *H. fowleri* within clade I instead of clade II. The distribution of *H. fowleri* suggests a complex pattern, with most registers from Mahury River (French Guyana) and few in drainages from the left bank of the Amazon River in Brazil (e.g., Maicuru River - our sampling site). This region in North Brazil harbors rivers that drain from the Guiana Shield and is a recognized region with poor knowledge on the ichthyofauna [44–46]. A similar pattern of distribution also occurs for the species *H. guianensis*, which is also placed in the clade I. Notably, *Harttia* species are usually restricted to small stretches of rivers with rocky or sandy bottoms and fast-flowing waters [47] and the Amazon River might serve as a great biogeographical barrier for species for clades I and II + III (Fig. 2), as widely recognized for several terrestrial and aquatic species [48–50].

A distinct sister-group relationship involving *H. dissidens* indicates more complex interspecific relationships than previously recognized. In our phylogeny, *H. dissidens* is sister to the *H. rondoni* + *H. villasboas* clade, with this group forming a sister lineage to the *H. duriventris*, and all these species are sister to *H. punctata*, the earliest diverging species in clade II at ~ 6 mya. In contrast, Londoño-Burbano and Reis [14] recovered *H. dissidens* as more ancestral to the whole clade II, while in Covain et al. [13], *H. dissidens* was placed as a sister to most species, with only *H. punctata* showing an older diversification. Besides, *Harttia* sp. 2 was recovered herein as deeply divergent from the remaining *Harttia* species, with an estimated split occurring ~ 25 Mya. This lineage exhibits a distinct karyotype, which is highly similar to that reported in *H. absaberi* [51], with 2n = 62 chromosomes and a high number of interstitial telomeric sites (ITS) [21]. SLMs are found in all *Harttia* species but *Harttia* sp. 2, supporting a distinction of the latter from the remaining *Harttia* species (Fig. 4). Indeed, *H. absaberi* was included in the molecular and morphological phylogeny presented by Cherobim [52], who recovered it as more closely related to other Loricariinae genera than to *Harttia*, suggesting it may warrant placement in a separate genus. Although our sampling included only a single outgroup (*Farlowella*), which diverged from *Harttia* ~ 30 Mya, the combined evidence from phylogenetic distance, divergence time, and cytogenetic data therefore supports the hypothesis that *Harttia* sp. 2, like *H. absaberi*, may not belong to *Harttia* sensu stricto.

Biogeographically, *Harttia* diversification mirrors Neotropical River evolution (Fig. 5a). The divergence of major clades aligns with the Miocene uplift of the Andes Mountain range that led to the reconfiguration of most drainage systems in South America, especially in the Amazonian region. The distribution of clade II species across formerly interconnected basins (e.g., Tocantins-Araguaia, Xingu, and Tapajós) reflects historical dispersal followed by vicariant speciation, a pattern supported by similar trends in other loricariid taxa and the highly similar ichthyofauna of those basins, as previously reported in other lineages such as *Cynolebias* spp., *Centromochlus schultzi*, and several Prochilodontidae [53–57]. Indeed, the redirection of river courses occurred in the late Miocene, especially proto-Tapajós, proto-Xingu, and proto-Tocantins-Araguaia [54], the development of mega-wetlands; and the formation of interfluves such as the Purus Arch and the Serra do Cachimbo possibly introduced new opportunities for both dispersal and vicariant isolation among freshwater organisms, particularly loricariids [58], which agrees with the patterns here observed for *Harttia*. Notably, patterns of biogeographical diversification seen in clade III coincide with the time when the uplift of the Serra do Mar and Serra da Mantiqueira mountain ranges occurred. These events have isolated certain portions of the La Plata Basin from the Atlantic South and Southeastern Drainages, as well as the São Francisco River Basin [58, 59], leading to vicariant processes among their similar ichthyofauna, as also observed for *Salminus* spp [60].

**Fig. 6:**
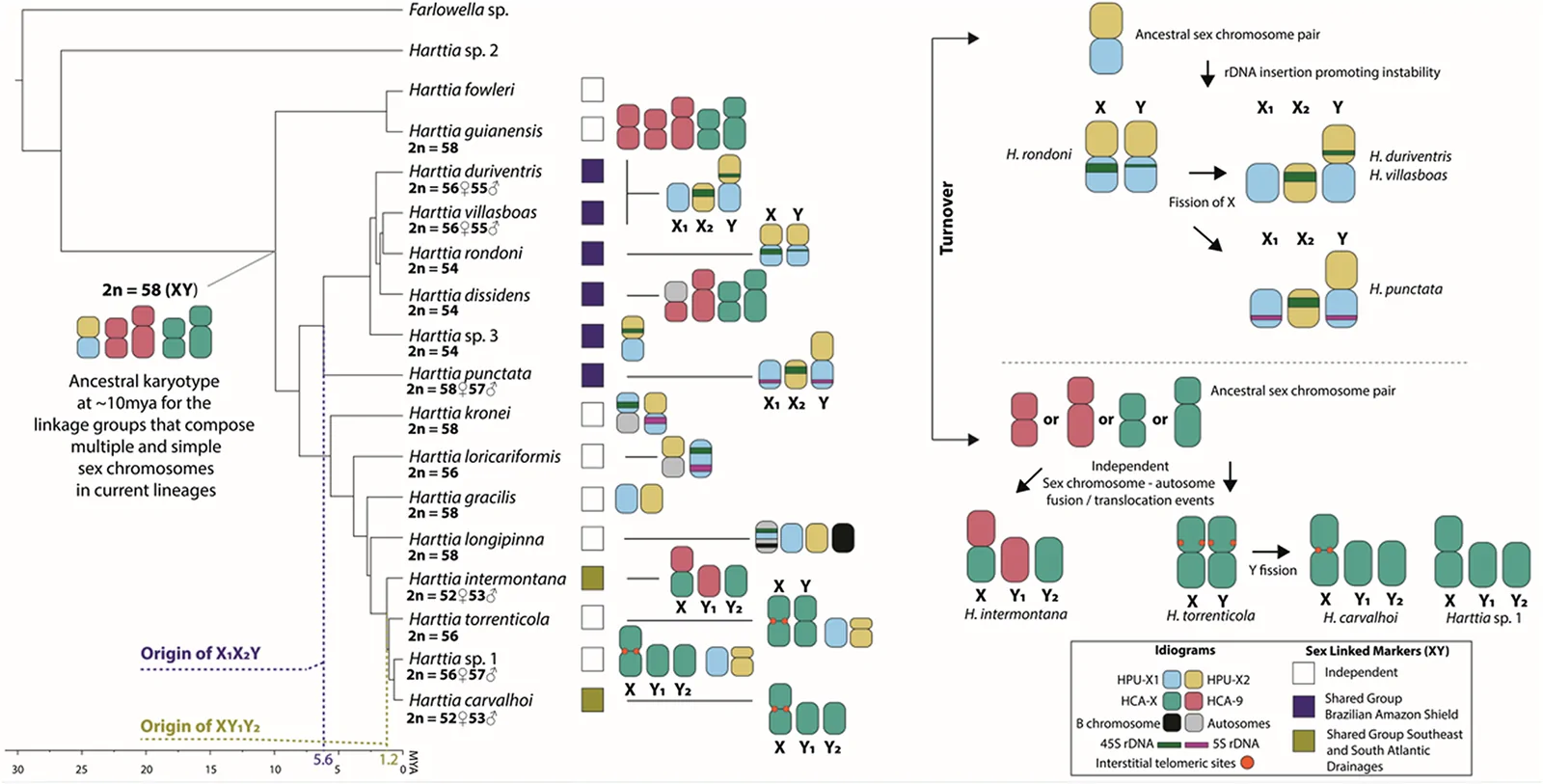
Rapid turnover of multiple sex chromosomes in *Harttia*. A compilation of previous cytogenetic data [18– [28, 61] and the results herein obtained with the main hypothesis for the sex chromosome evolution in *Harttia*. Chromosome colors follow the homology patterns established by Deon et al. [22, 23] and Sassi et al. [24, 28]. Idiograms are presented for each species-specific chromosome, while those pairs not shown in each species tip indicate the conservation of the ancestral state, as depicted on the left. Chromosomes are not drawn to scale

Our modeling approaches on both chromosome evolution (ChromEvol) and biogeographical patterns (BioGeoBEARS) support the ancestors of *Harttia* with a distribution in either the Eastern Amazon or Southeast/South Atlantic drainages. ChromEvol supported an ancestral karyotype of 2n = 58 chromosomes. Independently, integration of the SLM results with previous cytogenetic evidence reinforces the hypothesis that the ancestral *Harttia* lineage possessed a homomorphic XY sex chromosome system (Fig. 6). The findings thus corroborate earlier hypotheses based on comparative cytogenetics that proposed the same 2n as the ancestral state [21, 61]; therefore, reinforcing the hypothesis of independent reductions of karyotypes from 2n = 58 to 2n = 56 in *H. loricariformis*, *H. villasboas*, and *H. duriventris*, and subsequent reductions of 2n = 54 in *H. rondoni* and *Harttia* sp. 3 [23, 24]. These karyotype reductions were mostly facilitated by centric fusions, as indicated by our ChromEvol inference (Fig. 5b, Supplementary Data 3). Nevertheless, these chromosome rearrangements did not involve chromosomal components associated with the intricate multiple sex chromosome systems outlined in the genus [22, 24]. The dissociation of autosomal and sex chromosome dynamics underscores the intricate interaction of lineage-specific and conserved processes, such as Robertsonian translocations and inversions, that influence chromosome evolution in this group. Notably, breakpoints in *Harttia* are probably linked to the instability of rDNA loci [20–24, 28], particularly the 18S rDNA, which is not involved in the creation of the XY_1_Y_2_. Ribosomal sequences are known to form evolutionary breakpoint regions among loricariids [62, 63], as they increase genomic instability by facilitating the formation of double-strand breaks due to their high transcriptional activity [64, 65].

Our study states that recurrent turnover and independent evolution of multiple sex chromosomes are probably key mechanisms in the evolution of *Harttia*, reflecting the dynamic nature of sex chromosomes in this group. Across diverse lineages, including plants, fish, and reptiles, recurrent turnover of sex chromosomes within a relatively short evolutionary timescale has been reported [66–72], however, none is comparable with *Harttia*, where these events occur within a very short timeframe (~ 4 My). The arising of multiple sex chromosome systems usually results in postzygotic isolation, mainly due to segregation incompatibilities [7, 11, 73]. Rearrangements are also responsible for recombination suppression, which reduces gene flow near rearranged regions [74–76]. Furthermore, genes that contribute to reproductive isolation can accumulate on the neo-sex chromosomes, contributing to the speciation process [8, 77]. We hypothesize that a similar process may have happened on both rearrangement events, which resulted in XY_1_Y_2_ and X_1_X_2_Y systems, leading to faster reproductive isolation and speciation. This process was similarly documented in other animal lineages such as sticklebacks [8, 77] and was also proposed for other species such as *Brenthis* butterflies [78], African cichlids [79], and rock wallabies [80].

Among vertebrates, several mechanisms can drive sex chromosome turnover, including the acquisition of novel sex-determining mutations, translocations of pre-existing sex-determining (SD) genes, chromosomal fusions, and, in some cases, hybridization between populations with distinct SD systems [8, 11, 67, 81–86]. Given the absence of records for sympatric populations of *Harttia*, turnover driven by hybridization events appears unlikely. Instead, the emergence of the XY_1_Y_2_ system may be best explained by Y-autosome translocation, a mechanism widely reported in teleosts with multiple sex chromosome systems [11]. In contrast, the X_1_X_2_Y systems observed in clade II are more consistent with turnovers mediated either by novel SD mutations or translocations of ancestral SD genes. Notably, the association of ribosomal DNA (rDNA) sites with sex chromosomes in species of clade II suggests that rDNA insertions could act as catalysts for recombination suppression by breaking the linkage groups of SD.

To validate the presence of sex-linked markers, we cross-mapped the herein obtained SLMs to the chromosome-level genomes of *Pterygoplichtys* species (divergence from *Harttia* ~ 36 mya, according to TimeTree5 [87]). Overall, the results indicate that the *Harttia* sex-associated candidate markers have homologous sequences distributed across multiple chromosomes in *Pterygoplichthys*, and that there is no obvious one-to-one conservation of a single sex-associated chromosomal region between *Harttia* and these two *Pterygoplichthys* species. These results are therefore more consistent with genome-wide conservation of short marker sequences than with conservation of an ancestral sex chromosome, although establishing homology of larger chromosomal regions would require additional analyses such as synteny or whole-genome alignments. Notably, our PCA analysis of SLM (Supplementary Fig. 2) does not follow a clear pattern of clusterization related to sex chromosome systems, indicating a big cluster formed by most species and two independent clusters: one formed by *H. carvalhoi*, *H. torrenticola*, and *Harttia* sp. 1, and another by *H. intermontana*. This distribution indicates that most *Harttia* species share similarities regarding SLMs and that the XY_1_Y_2_ has an independent evolutionary pattern, also reinforced by the non-sharing SLMs (Fig. 4). The PCA and pairwise SLM-sharing analyses capture complementary properties of the dataset: the former summarizes similarity across the complete candidate SLM profile, whereas the latter identifies specific loci meeting the stringent sequence-sharing criterion. Thus, the PCA clustering should not be interpreted as evidence that the species share the same individual sex-linked loci or identical sex chromosome architecture. The SLM analyses provide comparative genomic evidence that complements, but does not independently reconstruct, the evolutionary history of *Harttia* sex chromosomes. Candidate SLMs were identified from a limited number of individuals per species and were subsequently compared among species and mapped to a distantly related loricariid genome. Consequently, shared SLMs provide evidence for conserved sex-associated genomic regions, whereas their absence cannot necessarily be interpreted as evidence of the absence of homology. Similarly, cross-species localization to *P. pardalis* chromosomes provides information about conserved genomic regions but cannot establish direct chromosomal correspondence because of the substantial phylogenetic distance and lineage-specific chromosomal rearrangements. Definitive reconstruction of sex chromosome evolution will require chromosome-level genomes and larger population samples for the relevant *Harttia* lineages.

In conclusion, our study presents the first genome-wide phylogenetic framework for the armored catfish genus *Harttia*, offering new insights into its evolutionary history, chromosomal evolution, and sex chromosome dynamics in teleosts. While broadly congruent with previous hypotheses, our phylogeny reveals several key topological differences, likely attributable to the increased resolution afforded by genome-wide data. Divergence time estimates highlight a relatively recent radiation of *Harttia* sensu stricto over the past 10 million years, tightly associated with major geological and hydrological transformations in South America driven by the formation of the Amazon River and the rise of the mountain ranges, and potentially accelerated by the rapid turnover of multiple sex chromosomes at 2.6 and 1.3 mya. Our results further support the hypothesis that *Harttia* sp. 2, like *H. absaberi*, falls outside *Harttia* sensu stricto, reflecting deep phylogenetic divergence corroborated by both cytogenetic and molecular evidence and challenging the monophyly of the genus. Chromosome evolution in *Harttia* is characterized by a dynamic interplay of variation in diploid numbers, lineage-specific reductions via centric fusions, and recurrent turnovers of sex chromosomes, leading to the independent origins of complex multiple sex chromosome systems that may have contributed to diversification in some *Harttia* lineages, although the causal relationship remains unresolved. Together, these findings show the presence of multiple sex chromosome systems and their association with elevated inferred speciation rates in the sampled *Harttia* lineages. It also reveals the intricate evolutionary processes shaping both genome structure and diversification in Neotropical fishes. Future studies incorporating broader taxonomic and genomic sampling will be essential to further refine the evolutionary history of *Harttia*, a remarkable repository of multiple sex chromosome systems.

## Methods

### Sampling

We selected all *Harttia* species with available cytogenetic data, following those described in Supplementary Table 1. The sex of the individuals was recovered from the morphological investigation of the gonads. Fish were anesthetized and euthanized with Eugenol, as approved by the Ethics Committee on Animal Experimentation of the Universidade Federal de São Carlos (Process number CEUA 7994170423). We have complied with all relevant ethical regulations for animal use.

### DArTseq and sex-linked markers

Liver tissue samples were collected from six individuals of each species (three males and three females), comprising a full dataset of 107 individuals, and submitted to Diversity Arrays Technology (DArT) Pty Ltd (University of Canberra, Canberra, Australia) for genotyping by sequencing. Briefly, DNA samples were digested with the restriction enzymes *PstI* and *SphI*, followed by adapter ligation and 30 cycles of PCR amplification. Amplified products were sequenced using single-end reads on an Illumina HiSeq2000 platform. Data filtering and quality control were performed by DArT Pty Ltd, excluding loci with reproducibility < 90% and read depths < 3.5× for SNPs and < 5× for Silico-DArT markers. For the characterization of sex-linked markers, both SNPs and Silico-DArT loci with missing data were not included in the analysis. For all remaining analyses (PCA, phylogenetic inference), loci with high frequencies of null alleles (call rate < 0.85) were filtered out.

Candidate sex-linked markers were identified following the pipeline proposed by Lambert et al. [37]. DArTseq datasets were first organized per species and sex, grouping males and females separately. For sex-linked marker characterization, datasets were analyzed independently for each species. Within each species, SNP and SilicoDArT loci containing missing genotypes among the six individuals were excluded prior to sex-linkage screening. For each individual, SNPs were tallied by heterozygous genotypes (coded as “2”) for each sex, using counts of homozygous genotypes (either “0” for the reference or “1” for the alternate allele) as the baseline. Given these criteria, sex-linked SNPs were defined as those in which all individuals of one sex had heterozygous genotypes and all individuals of the other sex had homozygous genotypes. On the other hand, sex-linked markers from Silico-DArT data (presence and absence) corresponded to those with the presence of the restriction recognition sites in all individuals from one sex but not the other.

The theoretical per-species probability (P) of a SNP randomly assorting by sex purely by chance is calculated as *P* = 2^(1–n)^ [37], where n is the number of individuals of the minority sex (here, *n* = 3 for all species). This gives *P* = 2^(1–3)^ = 0.25. However, the DArTseq™ protocol optimizes complexity-reduction chemistry at the sequencing plate level, meaning the same genomic regions are interrogated for all samples on a plate. It selects a representative portion of the dataset to verify which restriction enzymes better result in a complexity reduction for the whole species dataset. Considering that our SNP dataset comprised a total of 95,305 high-confidence markers derived from the same plate chemistry, we used a method to create a conservative genome-wide estimate of the false discovery rate by applying a Bonferroni correction for multiple testing across this entire SNP dataset (*N* = 95,305). The corrected per-test significance threshold was calculated as P_corrected = P/*N* = 0.25/95,305 ≈ 2.62×10⁻^6^. This extremely stringent threshold means that any SNP exhibiting fixed sex-associated differentiation in a species is unlikely to be a false positive. To assess the conservation and chromosomal distribution of the sex-linked markers identified herein, the complete set of markers was mapped against two chromosome-level *Pterygoplichthys* genome assemblies: *P. pardalis* (GCA_050231285.1; ASM5023128v1) and *P. disjunctivus* (GCA_059309555.1; ASM5930955v1). Marker sequences were aligned independently to each genome using BLASTn, and only alignments corresponding to the 26 chromosome sequences of each assembly were retained; unplaced scaffolds were excluded from subsequent analyses. BLAST hits were filtered using a minimum sequence identity of 95% and an alignment length of ≥ 30 bp. For each marker, the best-scoring chromosomal alignment was retained based on bitscore, thereby avoiding the representation of multiple redundant matches of the same marker within a genome. Genomic positions were calculated from the corresponding alignment coordinates, using the midpoint between the start and end positions of each retained alignment. The resulting distributions were plotted separately for *P. pardalis* and *P. disjunctivus* using R and ggplot2. To assess potential homology among sex-linked markers, multiple sequence alignments were performed in Geneious (Geneious Prime v2025.1, Dotmatics, Boston, USA) using the MUSCLE algorithm with default settings. Pairs of sequences exhibiting 100% similarity were identified from the pairwise distance matrix using a custom R script.

### Time-calibrated phylogeny and principal component analysis

We inferred the evolutionary history of *Harttia* species by Maximum Likelihood using RAxML-ng [88]. After filtering out SNPs under selection (5 sequences) using Bayescan [89], a total of 1,381 SNPs of 107 individuals from 18 species (17 *Harttia* + 1 *Farlowella*) were included in the analysis, totalizing 24,858 markers. All phylogenetic inferences were carried out with the full tag sequence, including invariant sites, rather than only with the SNPs. First, model selection was conducted in ModelTest-NG [90] with seed number defined as “12345”, and all models supported by RAxML-ng were tested. ModelTest-NG indicated HKY + I+G4 as the model with the smallest AIC (lnL − 118802.5778) and HKY + I as the model with the lowest BIC (lnL 240039.7218). The analysis was conducted with 50 initial maximum parsimony trees and 50 initial random trees. The analysis was performed with a total of 1000 bootstraps and mapped into the best tree. The tree with the highest log likelihood was used for time calibration. Finally, the tree was rooted using FigTree v.1.4.4 [91], defining *Farlowella* sp. as the outgroup.

Coalescent divergence times and population sizes were estimated under the Multispecies Coalescent Model using BPP [92] and calibrated to geological time with bppR [93]. BPP is comprehensive software for Bayesian Markov Chain Monte Carlo (MCMC) analysis, widely applied for species delimitation and divergence time estimation [92]. We first performed an A00 method analysis (no species delimitation and fixed tree topology). To ensure the consistency of the estimations in our dataset [94, 95], we performed independent runs with random subsets of different sizes (300, 400, and 500 marker), each with triplicates starting at distinct seed numbers. We assumed a mutation rate (µ) of 3 × 10^–9^ based on the inferred rate of the *Malapterurus electricus*, the closest Siluriformes with µ data [96]. We also adopted a divergence time estimation of 30 mya for *Harttia* and *Farlowella* based on the mean of estimates on TimeTree5 [97]. Considering these values, the theta prior was defined as an inverse gamma distribution of 3 0.002, and the tau prior was set as an inverse gamma distribution of 3 0.18. For this analysis, *Farlowella* was defined as the outgroup. Finetune was allowed, chain length was set to 400,000 generations, with a burn-in of 40,000, and sampling frequency was defined as 2. The first 15% of the samples of each run were discarded as burn-in, and each triplicate was concatenated. Effective sample size (ESS) values were checked on Tracer [91] to ensure that posterior distributions were adequately estimated. The final time calibrations were defined as the mean of the triplicate outputs with 95% HPD in bppR (Supplementary Table 2). We performed a time calibration using a prior node age, based on the mean divergence time of *Harttia* and *Farlowella* estimated on TimeTree5 [97]. To describe the temporal patterns of cladogenesis within the genus, we created a lineage-through-time plot (LTT plot) using ape 5.8 [98] on R.

Principal component analysis (PCA) was conducted using the R package dartR [99, 100]. Two distinct datasets were defined, one containing all sex-linked SNPs and a second using the whole SNP dataset, excluding the sex-linked ones (here defined as autosomal SNPs). The most representative PCs were selected for the plot, and the figure was constructed in Adobe Illustrator (Adobe Corporation).

### Character state-dependent branching

We applied the Binary State Speciation and Extinction (BiSSE) model [101] in R (4.3.1) with the package “hisse” [102] to test whether the presence of multiple sex chromosomes is associated with differences in diversification rates. The binary dataset for the character “multiple sex chromosomes” was defined with two states: absence = 0 and presence = 1. We followed the strategy proposed in Lambert et al. [103], to estimate state-specific speciation rates (λ₀ and λ₁), a single turnover rate (ε = µ/λ) applied to both states, and a single transition rate (q₀↔₁) between trait states. Likelihood optimization employed an initial simulated annealing phase followed by refinement using a subplex routine to obtain maximum likelihood estimates for speciation rates (λ₀, λ₁), a common turnover rate (ε), and transition rate (q₀↔₁). Confidence intervals for parameter estimates were derived through likelihood profiling via the SupportRegionHiSSE function, sampling 1000 points within the likelihood support region. To assess whether differences in speciation rates between trait states were statistically significant, we performed a likelihood ratio test (LRT). This involved comparing a full model where speciation rates were allowed to vary between states (λ₀ ≠ λ₁) to a constrained null model in which speciation rates were set equal across states (λ₀ = λ₁). The test statistics were calculated as twice the difference in log-likelihoods between the models, which was compared to a chi-squared distribution with one degree of freedom, revealing a p-value of 0.005559656.

### Chromosome number evolution

Chromosome counts and a time-calibrated phylogenetic tree with branch lengths were analyzed in ChromEvol v2.0 [104] to infer ancestral chromosome numbers. We evaluated six default models incorporating ascending dysploidy (single chromosome gains) and descending dysploidy (single chromosome losses) under a time-homogeneous framework. Model adequacy was assessed based on the best-fitting model (Dys). The minimum chromosome number was set to *n* = 26, corresponding to the lowest value observed in both *Harttia carvalhoi* and *H. intermontana*. The maximum chromosome number was set to *n* = 31, based on the highest diploid count (2n = 62) recorded for *H. absaberi* and *Harttia* sp. 2, the latter included in our dataset.

### Ancestral range estimation

The ancestral range of species was estimated using BioGeoBEARS [105], implemented in R. Three models of range evolution were tested: the dispersal–extinction–cladogenesis (DEC) model [106], the likelihood version of DIVA – DIVALIKE [107], and BAYAREALIKE [108]. Variants of these models incorporating a founder-event speciation parameter (“j”) were not considered, as this parameter has been widely criticized [109] and is unlikely to accurately represent dispersal in freshwater-restricted taxa where river connectivity is essential for gene flow.

Six freshwater ecoregions were defined based on the present-day distribution of species included in the analysis: Western Amazon (W), Eastern Amazon (E), Amazon Brazilian Shield (B), São Francisco and Northeastern drainages (F), Southern and Southeastern Atlantic drainages (A), and the La Plata basin (L). Species distributions were compiled from original descriptions and related literature [110] and cross-referenced with occurrence data obtained from GBIF, which were visualized in QGIS (Supplementary Fig. 5). Given the high endemism of *Harttia* species, which typically do not span multiple regions, we set the maximum number of areas allowed per node to two. This strategy avoids over-constraining the model, as using only a single area per node is not recommended [105].

To account for major historical changes in river connectivity, we defined three time slices based on Neotropical geological history, following frameworks established by Conde-Saldaña et al. [111]: >23 Mya, 23–10 Mya, and < 10 Mya. These periods encompass key events in river capture and paleodrainage evolution of the Neotropical region [54, 112–114]. The map representing these connections was created using QGIS 3.28.2-Firenze using merged ecoregions from Abell et al. [115].

### Statistics and reproducibility

All statistical analyses were performed as described in the Methods. We employed standard statistical practices, including multiple-testing correction (Bonferroni) for marker discovery, model selection (AIC/BIC) for phylogenetics, and likelihood-based hypothesis testing (BiSSE). Bayesian methods were checked for convergence (ESS > 200). Sample sizes for each experiment are provided in the relevant Methods subsections. All custom code used for data analysis, figure generation, and statistical testing is publicly available on GitHub (see Data Availability statement), ensuring the full reproducibility of this work.

### Structure analysis

The *structure* [116] analyses were performed using a burn-in period of 30,000 and a total of 200,000 chains for each run. Ploidy was set as 2. For each K, a total of ten independent runs were performed. Runs were performed under an admixture model, with no linkage and no locprior information. A custom script was designed to generate random seed numbers for each run and parallelize the analysis.

The full dataset (107 individuals, 1,375 markers) runs were performed from K = 1 to K = 19. After all runs, the plots were produced with Clumpak [117] through the StructureSelector web server [118]. To evaluate the best values of K, we have applied different methods, including the Evanno method [119], the Puechmaille method [120], and the Choose K method [121]. All STRUCTURE plots are presented in Supplementary Data 4.

## Supplementary Information

Below is the link to the electronic supplementary material.

Supplementary Material 1

Supplementary Material 2

Supplementary Material 3

Supplementary Material 4

Supplementary Material 5

## Data Availability

No datasets were generated or analysed during the current study.
