# Supplementary material for: Rapid turnover of sex chromosomes is associated with speciation in Neotropical armored catfish Harttia (Siluriformes, Loricariidae)
Source: Biol Direct. 2026 Oct 21;21:211. doi: 10.1186/s13062-026-00994-y (PMC13685301; doi:10.1186/s13062-026-00994-y)
Supplement: Supplementary file 1 [file 13062_2026_994_MOESM5_ESM.pdf]

Major modes for the uploaded data:

sp_RP = H. sp. 3
sp_RM = H. sp. 1
sp_BG = H. sp. 2

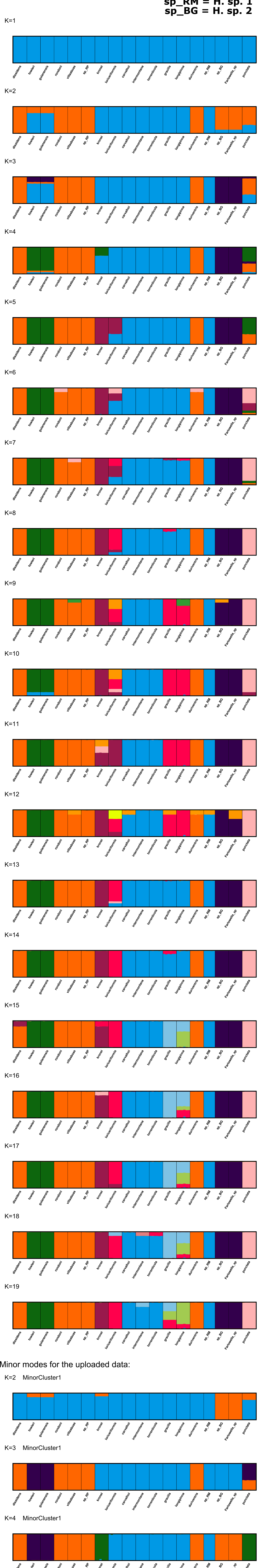

Minor modes for the uploaded data:

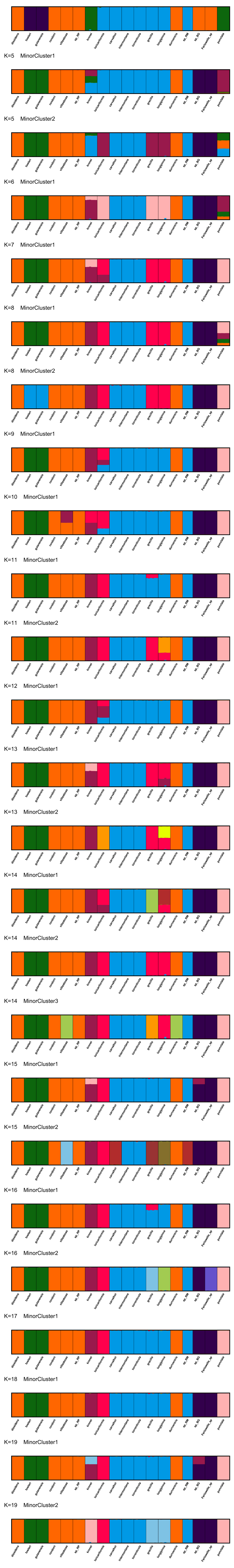

Division of runs by mode:

K=1	10/10
K=2	8/10, 2/10
K=3	9/10, 1/10
K=4	9/10, 1/10
K=5	4/10, 4/10, 2/10
K=6	7/10, 3/10
K=7	7/10, 3/10
K=8	5/10, 4/10, 1/10
K=9	8/10, 2/10
K=10	8/10, 2/10
K=11	4/10, 3/10, 3/10
K=12	6/10, 4/10
K=13	5/10, 3/10, 2/10
K=14	4/10, 3/10, 2/10, 1/10
K=15	5/10, 4/10, 1/10
K=16	7/10, 2/10, 1/10
K=17	7/10, 3/10
K=18	7/10, 3/10
K=19	6/10, 3/10, 1/10

Major modes for the uploaded data:

sp_RP = H. sp. 3
sp_RM = H. sp. 1
sp_BG = H. sp. 2

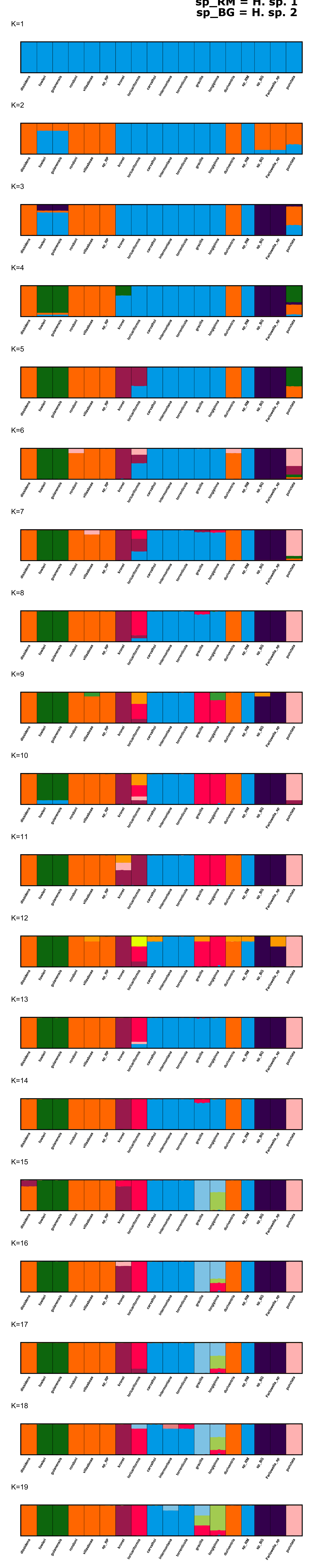

Minor modes for the uploaded data:

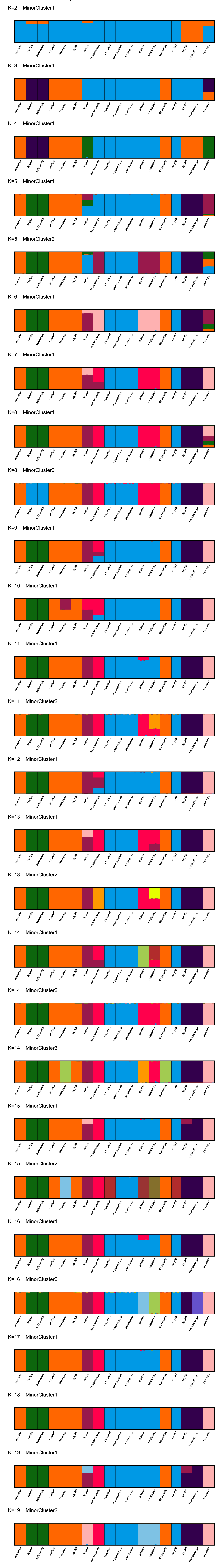

Division of runs by mode:

K=1 10/10
K=2 8/10, 2/10
K=3 9/10, 1/10
K=4 9/10, 1/10
K=5 4/10, 4/10, 2/10
K=6 7/10, 3/10
K=7 7/10, 3/10
K=8 5/10, 4/10, 1/10
K=9 8/10, 2/10
K=10 8/10, 2/10
K=11 4/10, 3/10, 3/10
K=12 6/10, 4/10
K=13 5/10, 3/10, 2/10
K=14 4/10, 3/10, 2/10, 1/10
K=15 5/10, 4/10, 1/10
K=16 7/10, 2/10, 1/10
K=17 7/10, 3/10
K=18 7/10, 3/10
K=19 6/10, 3/10, 1/10
