# Supplementary material for: Rapid turnover of sex chromosomes is associated with speciation in Neotropical armored catfish Harttia (Siluriformes, Loricariidae)
Source: Biol Direct. 2026 Oct 21;21:211. doi: 10.1186/s13062-026-00994-y (PMC13685301; doi:10.1186/s13062-026-00994-y)
Supplement: Supplementary file 2 [file 13062_2026_994_MOESM4_ESM.docx]

**Supplementary Information**

**Rapid turnover of sex chromosomes is associated with speciation in Neotropical armored catfish *Harttia* (Siluriformes, Loricariidae)**

Francisco de M. C. Sassi^1, 2, 3^, Fernando Henrique S. de Souza^2, 4^, Manolo F. Perez^5^, Foyez Shams^3^, Tariq Ezaz^3^, Geize A. Deon^2, 3^, Thomas Liehr^6^*, Luohao Xu^1^, Marcelo de B. Cioffi^2^

**Supplementary Table 1**. Species, sampling sites, number and sex of individuals (N), sex chromosome system (SCS), and reference to the cytogenetic data. The numbers before the species name correspond to those in **Figure 1**.

| **Species** | **Sampling sites** | **N** | **SCS** | **Reference** |
| --- | --- | --- | --- | --- |
| 1 - *Farlowella* sp. | Triângulo farm,  Pontes e Lacerda – MT (15°10'43.7"S 59°17'01.7"W) | 3♀, 3♂ |  | - |
| 2 - *Harttia carvalhoi* | Ribeirão Grande stream, Pindamonhangaba – SP (22°46'2.98''S 45°26'7.05''W) | 3♀, 3♂ | XY_1_Y_2_ | Centofante et al., 2006 |
| 3 - *Harttia dissidens* | Tambor stream, Rurópolis – PA (4°5'37.8''S 55°0'30.2''W) | 3♀, 3♂ |  | Sassi et al., 2021 |
| 4 - *Harttia duriventris* | Parauapebas River, Canaã dos Carajás – PA (6°30'06.5''S 50°02'35.3''W) | 3♀, 3♂ | X_1_X_2_Y | Sassi et al., 2020 |
| 5 - *Harttia fowleri* | Maicuru river, Alenquer – PA (1°36'32.3"S 54°22'28.5"W) | 3♀, 3♂ |  | - |
| 6 - *Harttia gracilis* | Machadinho stream, Santo Antônio do Pinhal – MG (22°48'31"S 45°41'21"W) | 3♀, 3♂ |  | Blanco et al., 2017 |
| 7 - *Harttia guianensis* | Paraíso stream, Alenquer – PA (1°29'02.2''S 54°50'31.2''W) | 3♀, 3♂ |  | Sassi et al., 2021 |
| 8 - *Harttia intermontana* | Piranga river, Carandaí – MG (20°59'34.0''S 43°43'30.0''W) | 3♀, 3♂ | XY_1_Y_2_ | Deon et al., 2020 |
| 9 - *Harttia kronei* | Açungui river, Campo Largo – PR (25°22'44"S 49°39'08"W) | 3♀, 3♂ |  | Alves et al., 2003 |
| 10 - *Harttia longipinna* | São Francisco river, Pirapora – MG (17°21'22.8"S 44°51'0.2"W) | 3♀, 3♂ |  | Blanco et al., 2012 |
| 11 - *Harttia loricariformis* | Paraitinga river, Silveiras – SP (22°52.225'S 44°51.041'W) | 3♀, 3♂ |  | Kavalco et al., 2004 |
| 12 - *Harttia punctata* | Itiquira river, Formosa – GO (15°19'25''S 47°25'26''W) | 3♀, 3♂ | X_1_X_2_Y | Blanco et al., 2014 |
| 13 - *Harttia rondoni* | 13 de Maio River, Cachoeira da Serra – PA (8°38'53.0''S 55°01'41.0''W) | 3♀, 3♂ | XY | Sassi et al., 2020 |
| 14 - *Harttia* *torrenticola* | Araras stream, Piumhi – MG (20°26'16.4''S 45°55'39.8''W) | 3♀, 3♂ | XY | Blanco et al., 2013 |
| 15 - *Harttia villasboas* | Curuá River, Cachoeira da Serra – PA (8°44'09.0"S 54°57'46.0"W) | 3♀, 3♂ | X_1_X_2_Y | Sassi et al., 2020 |
| 16 - *Harttia* sp. 1 | Macacos stream, Silveiras – SP (22°40'43''S 44°51'25''W) | 3♀, 3♂ | XY_1_Y_2_ | Deon et al., 2020 |
| 17 - *Harttia* sp. 2 | Barra Grande river, Prudentópolis – PR (24°58'40.72''S 51°7'34.25''W) | 3♀, 3♂ |  | Deon et al., 2020 |
| 18 - *Harttia* sp. 3 | Rio do Peixe, Cachoeira da Serra, Altamira – PA (08°39'20.7''S 55°09'24.1''W) | 3♀, 3♂ |  | Sassi et al., 2021 |

**Supplementary Table 2**. Time-calibration mean values in million years for the divergence of the clades seen in **Figure 1** obtained from bppR. Names of clades correspond to those in **Supplementary Figure 1**. Mean represents the total mean for the triplicate, while MeanLower and MeanUpper represent the mean for the lower and upper values indicated in each triplicate.

| **Clade** | **Mean** | **MeanLower** | **MeanUpper** | **Clade Description** |
| --- | --- | --- | --- | --- |
| t_19 | 29.5735748177394 | 28.3388079850934 | 30.8083416503854 | *Farlowella* crown age |
| t_20 | 26.2839570159164 | 22.0097412188964 | 30.5581728129364 | *Harttia* sp. 2 crown age |
| t_21 | 10.0482010342951 | 7.41775198568184 | 12.6786500829083 | Crown age of *Harttia* stricto sensu |
| t_22 | 7.87160550285082 | 4.75290779877554 | 10.9903032069261 | Split between clades II and III |
| t_23 | 5.83463939970166 | 3.0664037175818 | 8.60287508182152 | Crown age of kronei |
| t_24 | 3.92206054974575 | 2.53805835066998 | 5.30606274882152 | Crown age of loricariformis |
| t_25 | 2.83239189070291 | 1.90211861562663 | 3.76266516577919 | Crown age of gracilis |
| t_26 | 2.55017237440836 | 1.74143466787055 | 3.35891008094616 | Crown age of longipinna |
| t_27 | 1.26138619679288 | 0.820443943363059 | 1.70232845022271 | Crown age of intermontana |
| t_28 | 1.09927130824559 | 0.71521103239904 | 1.48333158409214 | Crown age of torrrenticola |
| t_29 | 0.701585160692601 | 0.309333411893242 | 1.09383690949196 | Crown age of sp. 1 - carvalhoi |
| t_30 | 6.3070841381895 | 3.72632349394238 | 8.88784478243661 | Crown age of punctata |
| t_31 | 2.5888160062289 | 1.73231238323071 | 3.44531962922708 | Crown age of sp. 3 |
| t_32 | 2.12344482242399 | 1.39685631127116 | 2.85003333357682 | Crown age of duriventris |
| t_33 | 1.80427277602175 | 1.16092980964123 | 2.44761574240227 | Crown age of dissidens |
| t_34 | 1.5787068681203 | 0.981421700324859 | 2.17599203591574 | Crown age of villasboas - rondoni |
| t_35 | 1.31731678110928 | 0.498872325901989 | 2.13576123631658 | Crown age of guianensis-fowleri |

**Supplementary Table 3.** Summary of shared sex-linked markers, indicating the species that share those sequences and which class (SNP or SilicoDArT) and heterogamety - **H** (XY or ZW) they indicate.

| **Species** | **Shared Sequences** | **Type** | **H** |
| --- | --- | --- | --- |
| *Harttia carvalhoi* | TGCAGACAAACAGCAATGTTCTTTTTGAAGAGCGATGGCTTTCTCCATGCAACCCTGCTATGCATGAGC | SNP | XY |
| *Harttia intermontana* | TGCAGGTTCTTCTCTGATAGCCGCATG | SNP | XY |
|  | TGCAGGCATACTGTACGTGAGAGAAGAACGGAGATCAAGTTGATCAGAATTCAGTCTGCATG | SilicoDArT | XY |
|  | TGCAGTCTCTAACTGTAGAGTTAAGAGTACAAAGTGCACCTATATGGTAAGTGGAGCTGATAAAATGAT | SilicoDArT | XY |
|  | TGCAGCTCATAGCACCGCCCCCACAGGTGGTGACCCTTTTTTGGCTAGGCAGTGTATATATAAGTTTTG | SilicoDArT | XY |
|  | TGCAGTTATCTGTTTATTCAAGTGGTCATGTAAATAGCATACTCTGATTTGGCAGATTGGATTAATGCC | SilicoDArT | XY |
|  | TGCAGTAGGAATGATGGTATTTACATGTATCTGGTAGGAGCACCAACGCTGCCCAGCCGGCCACTAGAG | SilicoDArT | XY |
|  | TGCAGTCTTGCCTGCGTGGTCAACCTTAATTAACATCCAAAACTAGACTCAACATGAAACTAAAAGAAA | SilicoDArT | XY |
|  | TGCAGCCATGCGTTTAGTGTTTAGGCCTTTTGGCTCGGTACCTGTGTGTGTGTGTGTGTGTGTGTGTGT | SilicoDArT | XY |
|  | TGCAGGTGAAACCCTAAAATGTGACACACACGAGACAACAGCAAATAACAAACAGAATTTAACATTTTT | SilicoDArT | XY |
|  | TGCAGGAGGGAATGAGCGCTTAAGTGATGTGCCGCTGAGGGAAGCACATGCGGCCAGCAGATAAAGCAG | SilicoDArT | XY |
|  | TGCAGTGCAGCAGTGCCAATATCTCACATTATTGCACAAACCTTGGATGCGTTATTTAGACATTTTTCC | SilicoDArT | XY |
|  | TGCAGAATTCTGTAATTCACAGTATGCTAAGTGAATATTGATGTACTTGTACCACAATGTGTAAGCATG | SilicoDArT | XY |
|  | TGCAGTAATGCTTGAGTAGATGGTACTGGTCAAAGTAACATCCACATGAATGCCAGGACTCTAGTTCTC | SilicoDArT | XY |
|  | TGCAGATTTAAGCAACGGTAACTGTAACTGGTTTGAATTTTAAACAACAGACAATGCACAAACAGGTCT | SilicoDArT | XY |
|  | TGCAGCCAAGAAAGCTGAGAAGAAGCACTGGAAAAGGAACCCAGAGAGAAACTGTGATGTGAGCATCTT | SilicoDArT | XY |
|  | TGCAGAGCCATCCAACTACCTTGGTGACTTCATTCAAGGAAATGGATAAAGCAGGCATG | SilicoDArT | XY |
|  | TGCAGATTTTTATTTGATAAAATTTTTTAAAACCAGGTATAATTTTCTTTACACTAAAATGTCATGAAG | SilicoDArT | XY |
|  | TGCAGTGTGAGTCCACATTCGTGTTAGACTTGGTCTACCTATGGCCAGGGTAGGACTTTAAGTTTCACT | SilicoDArT | XY |
|  | TGCAGTGACTGGTTATATCCCAGTAACACACCGTTGAAATGCATG | SilicoDArT | XY |
|  | TGCAGCCATCACTAAAACTCATTCAGACATTCAGGATTAGAACTCGATTAAAACTTTTCCCTCTATTGG | SilicoDArT | XY |
|  | TGCAGATCACACACAAATCAGATTATTTACACACACACACCGCCATACAGATCTTACTAATTTTTCCAC | SilicoDArT | XY |
| *Harttia dissidens* | TGCAGTCTCTAATTGTAGAACTACAAAGTGCAAATATTTTCCTATATATTTTCATGGGACAACACTATA | SilicoDArT | XY |
| *Harttia rondoni* |  |  |  |
|  |  |  |  |
| *Harttia duriventris* | TGCAGGTTACAACAGTCACCAAACAGTAGGAAGTGTGCATG | SilicoDArT | XY |
| *Harttia rondoni* |  |  |  |
|  |  |  |  |
| *Harttia villasboas* | TGCAGGTAAATACAACAGAGAACATACACGCATG | SNP | XY |
| *Harttia rondoni* | TGCAGGTTCATTTCTTGATGTTTATGGACCACGTGGCCGAATATAAGGTGGCCTTAAATAAAGCATGAG | SilicoDArT | XY |
| *Harttia* sp. 3 |  |  |  |
| *Harttia dissidens* | TGCAGCTCTATGAGGACGGGCTCAACGTTCTCATTACTATGTCTCTGAGAATTAGTAGGGTCACCTCTC | SilicoDArT | XY |
| *Harttia duriventris* | TGCAGATGTGCTGAAGTCATTCAAAGCAAAGGCATG | SilicoDArT | XY |
| *Harttia villasboas* | TGCAGTGATCTGGTGACTAAGGGTTGGTTCCCAACTTGAGCGCCATGCTGCTGGATATGACCCATCCAA | SilicoDArT | XY |
|  | TGCAGGGGTTAGTGCTCTCGTCTAACGGTTCCAGTAACCCATGTCTGAGTTCTGGCCTGAGCACTCCCT | SilicoDArT | XY |
| *Harttia duriventris* | TGCAGTACTCCTTAACTACAACTGACTGCATATCAGTTCCTCTTTTCATGTCCTGTATAGTTCGCCTGT | SilicoDArT | XY |
| *Harttia villasboas* |  |  |  |
| *Harttia* sp. 3 |  |  |  |
|  |  |  |  |
| *Harttia dissidens* | TGCAGGTAAATACAACAGAGAACATGCACGCATG | SilicoDArT | XY |
| *Harttia duriventris* |  |  |  |
| *Harttia punctata* |  |  |  |
|  |  |  |  |
| *Harttia dissidens* | TGCAGAACCAACTCCATGTCTCCACGATCCTCTCAGTCTCCACCAGCCCTCTAGTATTTCTGCTACAGA | SilicoDArT | XY |
| *Harttia duriventris* |  |  |  |
| *Harttia rondoni* |  |  |  |
| *Harttia villasboas* |  |  |  |
|  |  |  |  |
| *Harttia dissidens* | TGCAGCATATATGCAAATATGTGGGAGGAATGTATGAGAAAACTGGAATACCTGGAAAAAAACTCAAGC | SilicoDArT | XY |
| *Harttia rondoni* |  |  |  |
| *Harttia villasboas* |  |  |  |
| *Harttia* sp. 3 |  |  |  |
|  |  |  |  |
| *Harttia dissidens* | TGCAGCAACCTGATAGTAGAAATGTTCAATAGGTGCCTTGCAGCCAGATGCAGCATG | SilicoDArT | XY |
| *Harttia duriventris* |  |  |  |
| *Harttia punctata* |  |  |  |
| *Harttia villasboas* |  |  |  |
| *Harttia* sp. 3 |  |  |  |

**Supplementary Figure 1**. Maximum likelihood tree showing all individuals investigated for each species.

**Supplementary Figure 2**. PCA plot of autosomal and sex-linked SNP marker data. Clusters present the geographic distribution of the species, with blue for the Amazon Brazilian Shield, red for the Guyana Shield, and gray for South and Southeastern Atlantic Drainages and La Plata. The legend on the right represents each species with a colored circle and a number. The sex of the individuals follows circles for females and triangles for males. Homologous sex chromosomes are denoted in the legend using the same colors.

**Supplementary Figure 3**. Biogeographic modeling on BioGeoBEARS using the DEC model.

**Supplementary Figure 4**. Occurrences of *Harttia* species gathered from GBIF, plotted using QGIS 3.28.2 - Firenze and Natural Earth.

**Supplementary Figure 5**. Evanno method for STRUCTURE.

**Supplementary Figure 6**. Puechmaille method for evaluating the STRUCTURE results.
